# Relationship between surgical R0 resectability and findings of peripancreatic vascular invasion on CT imaging after neoadjuvant S-1 and concurrent radiotherapy in patients with borderline resectable pancreatic cancer

**DOI:** 10.1186/s12885-020-07698-0

**Published:** 2020-12-02

**Authors:** Sho Yasuta, Tatsushi Kobayashi, Hidetoshi Aizawa, Shinichiro Takahashi, Masafumi Ikeda, Masaru Konishi, Motohiro Kojima, Hirofumi Kuno, Katsuhiko Uesaka, Soichiro Morinaga, Atsushi Miyamoto, Hirochika Toyama, Norihisa Takakura, Keishi Sugimachi, Wataru Takayama

**Affiliations:** 1 Department of Hepato-biliary Pancreatic Surgery, National Cancer Center Hospital East, Kashiwa, Japan; 2 Department of Diagnostic Radiology, National Cancer Center Hospital East, 6-5-1 Kashiwanoha, Kashiwa, Chiba, 277-8577 Japan; 3 Department of Surgery, Jichi Medical University Saitama Medical Center, Saitama, Japan; 4 Department of Hepato-biliary Pancreatic Oncology, National Cancer Center Hospital East, Kashiwa, Japan; 5 Division of Pathology, National Cancer Center Hospital East, Kashiwa, Japan; 6 Department of Hepato-biliary Pancreatic Surgery, Shizuoka Cancer Center Hospital, Shizuoka, Japan; 7 Department of Hepato-Biliary and Pancreatic Surgery, Kanagawa Cancer Center, Yokohama, Japan; 8 Department of Hepato-Biliary-Pancreatic Surgery, National Hospital Organization Osaka National Hospital, Osaka, Japan; 9 Department of Hepato-Biliary-Pancreatic Surgery, Kobe University Graduate School of Medicine, Kobe, Japan; 10 Department of Surgery, Fukuyama City Hospital, Fukuyama, Japan; 11 Department of Hepato-Biliary-Pancreatic Surgery, National Hospital Organization Kyusyu Cancer Center, Fukuoka, Japan; 12 Department of Hepato-Biliary-Pancreatic Surgery, Chiba Cancer Center, Chiba, Japan

**Keywords:** Borderline resectable pancreatic cancer, Neoadjuvant chemoradiotherapy, S-1 and concurrent radiotherapy, Progression of vascular invasion, R0 resectability, JASPAC 05

## Abstract

**Background:**

Borderline resectable pancreatic cancer (BRPC) is frequently associated with positive surgical margins and a poor prognosis because the tumor is in contact with major vessels. This study evaluated the relationship between the margin-negative (R0) resection rate and findings indicating peripancreatic vascular invasion on multidetector computed tomography (MDCT) imaging after neoadjuvant chemoradiotherapy (NACRT) in patients with BRPC.

**Methods:**

Twenty-nine BRPC patients who underwent laparotomy after neoadjuvant S-1 with concurrent radiotherapy were studied retrospectively. Peripancreatic major vessel invasion was evaluated based on the length of tumor-vessel contact on MDCT. The R0 resection rates were compared between the progression of vascular invasion (PVI) group and the non-progression of vascular invasion (NVI) group.

**Results:**

There were 3 patients with partial responses (10%), 25 with stable disease (86%), and 1 with progressive disease (3%) according to the RECISTv1.1 criteria. Regarding vascular invasion, 9 patients (31%) were classified as having PVI, and 20 patients (69%) were classified as having NVI. Of the 29 patients, 27 (93%) received an R0 resection, and all the PVI patients received an R0 resection (9/9; R0 resection rate = 100%) while 90% (18/20) of the NVI patients underwent an R0 resection. The exact 95% confidence interval of risk difference between those R0 resection rates was − 10.0% [− 31.7–20.4%].

**Conclusions:**

Patients with BRPC after NACRT achieved high R0 resection rates regardless of the vascular invasion status. BRPC patients can undergo R0 resections unless progressive disease is observed after NACRT.

**Trial registration:**

UMIN-CTR, UMIN000009172. Registered 23 October 2012

## Background

Pancreatic cancer is highly lethal and has an extremely poor prognosis [1, 2]. Previous reports have shown a median survival time of 15.2 months after curative-intent resection and 6–8 months without surgery [1, 3]. Surgical resection is the only possible cure, but most patients are diagnosed at an advanced stage, and only 15–20% of patients are considered candidates for curative-intent resection [4]. For patients undergoing curative-intent resection, margin-positive (R1) residual tumor resection is significantly correlated with a poorer survival outcome [5].

Borderline resectable pancreatic cancer (BRPC) is frequently associated with positive surgical margins and a poor prognosis because the tumor is in contact with major vessels. According to the National Comprehensive Cancer Network (NCCN) guidelines, neoadjuvant chemotherapy (NAC) or neoadjuvant chemoradiotherapy (NACRT) is recommended for patients with BRPC [6, 7]. Neoadjuvant therapy may assist in shrinking the primary tumor, controlling micrometastases, and identifying patients at risk of early disease progression before surgery [8, 9]. Moreover, neoadjuvant therapy may improve the rate of R0 resection, which is a strong prognostic factor in BRPC patients undergoing pancreatic resection. Although several clinical trials using either NAC or NACRT have been conducted for BRPC patients, a standard treatment for BRPC has not been established. Recently, a phase II multicenter prospective trial (JASPAC 05) elucidated the efficacy and feasibility of neoadjuvant S-1 and concurrent radiotherapy at increasing the R0 resection rate and improving survival in BRPC [10].

Whether surgery is indicated for patients with localized pancreatic cancer is usually assessed according to the degree of tumor-vessel contact measured using multidetector computed tomography (MDCT). However, in BRPC patients who have received NACRT, the decision to proceed with surgery can be difficult because the MDCT findings do not always correspond with the actual resectability, partly because of inflammatory change caused by NACRT. Some of our patients in whom the primary tumor had shrunk but the perivascular component of the tumor had paradoxically progressed after NACRT were able to receive an R0 resection [11]. Based on the above-mentioned experiences, we began to presume that the radiological progression of the perivascular component of the tumor after NACRT might not correspond with the actual progression if the primary tumor does not show signs of progression. If so, R0 resectability after NACRT should be evaluated according to not only the degree of tumor-vessel contact, but the radiological response of the primary tumor.

We tested this hypothesis by evaluating the R0 resection rates according to the combinations of radiological changes in the perivascular tumor and the tumor response in patients who had undergone a laparotomy after receiving neoadjuvant S-1 and concurrent radiotherapy, including the patients enrolled in the JASPAC 05 trial. The relationship between the perivascular tumor response and clinicopathological variables and the factors associated with the pathological tumor response were also examined.

## Methods

### Patients and clinicopathological data collection

This was a retrospective observational study. Patients who were either enrolled in the JASPAC 05 trial or received NACRT with S-1 for BRPC at the National Cancer Center Hospital East (NCCHE) between 2008 and 2017 were examined. The patients were included in the present study if a laparotomy was conducted for curative-intent after NACRT. Patients who were found to have distant metastasis at the time of laparotomy were excluded. Among the 14 institutions that enrolled patients in JASPAC 05, 8 institutions agreed to collaborate with the present study. As a result, 24 patients with BRPC who had participated in the JASPAC 05 trial and 5 patients with BRPC who had received NACRT at the NCCHE were included. All 29 patients had a cytologically or histologically confirmed diagnosis of pancreatic ductal adenocarcinoma prior to undergoing NACRT.

Clinicopathological data were retrieved from the JASPAC 05 data and the medical records of the patients who were treated at the NCCHE. Patient characteristics including age, sex, Eastern Cooperative Oncology Group (ECOG) performance status, tumor location, clinical staging as per the Union for International Cancer Control (UICC) classification (7th edition) [12], and type of tumor-vessel contact before NACRT were noted. Tumor size on CT imaging, the carcinoembryonic antigen (CEA) level, and the carbohydrate antigen 19–9 (CA 19–9) level were analyzed before and after NACRT. The tumor response was evaluated according to the response evaluation criteria in solid tumors classification (RECIST) v1.1 [13]. Intraoperative variables including the type of surgical procedure, portal vein resection, partial resection of the superior mesenteric artery (SMA) plexus, the number of retrieved lymph nodes, and blood loss were also examined.

Pathological findings including the tumor size, lymph node metastasis, portal vein invasion, plexus invasion, pathological staging as per the UICC classification (7th edition), resection margin, and pathological response were noted. R0 resection was defined as the absence of tumor cells present at the resection margin. The pathological response for NACRT was evaluated using the Evans classification [14]. The pathological response was classified as follows: grade I < 10% or no tumor cell destruction; grade IIa, destruction of 10–50% of tumor cells; grade IIb, destruction of 51–90% of tumor cells; grade III, < 10% viable-appearing tumor cells; and grade IV, no viable cells. A pathologist at each institution evaluated the histological examination results.

### Neoadjuvant chemoradiotherapy

The NACRT regimen consisted of S-1 with concurrent radiotherapy, as reported previously [15]. Briefly, a total dose of 50.4 Gy was delivered in 28 fractions over 5.5 weeks. S-1 was administered orally at 40 mg/m^2^ twice daily for 28 consecutive days, followed by a 14-day rest period per course on the day of irradiation.

### Surgical indication after NACRT

Patients who met the surgery-entry criteria, consisting of the absence of distant metastasis, no local progression precluding an R0 or R1 resection, a good performance status (PS 0/1), no massive ascites, no massive pleural effusion, no serious infection, no serious adverse NACRT events, and adequate organ system function, underwent surgery 15 to 56 days after the end of NACRT.

When diagnosing local progression precluding R0 or R1 resection, the occurrence of progressive disease (PD) according to the RECIST was basically used as our consensus-based standards for the criteria, while 1 patient with PD who conducted laparotomy at the NCCHE because of patient’s strong preference was included in the study. The perivascular findings obtained using MDCT after NACRT were not taken into consideration when determining the surgical indications unless the tumor response was PD.

### Diagnosis and classification of CT imaging findings

BRPC was diagnosed via a 16- or 64-slice MDCT with a pancreas protocol including triphasic cross-sectional imaging with a 2-mm slice thickness. BRPC was defined as bilateral impingement on the superior mesenteric vein or portal vein (BR-PV) or tumor contact ≤180° with the superior mesenteric artery (BR-SMA), the common hepatic artery (BR-CHA), or the celiac artery (BR-CeA) based on the NCCN 2009 guidelines [6].

Two radiologists (T.K. and H.K.) who were blinded to the patients’ clinical histories retrospectively analyzed the CT images before and after NACRT. The radiological response of the perivascular component of the tumor was evaluated and graded as peripancreatic major vessel invasion. The response of the peripancreatic major vessel invasion was graded according to the change in the longitudinal length of tumor-vessel contact. The progression or the shrinkage of peripancreatic major vessel invasion was defined as an increase or decrease in the length of tumor-vessel contact ≥2 mm. Otherwise, the peripancreatic major vessel invasion was graded as stable. Patients were classified into 2 groups: progression of vascular invasion (PVI, progression) or non-progression of vascular invasion (NVI, shrinkage or stable).

The R0 resection rates according to the various combinations of peripancreatic major vessel invasion response and tumor response were analyzed. The relationships between the peripancreatic major vessel invasion response and other evaluation items such as the rates of reduction in CEA and CA 19–9 after NACRT, the tumor response according to the RECIST, and the pathological response according to the Evans classification were evaluated. Factors associated with a pathological response of Evans grade ≥ IIb were also analyzed.

### Statistical analysis

Categorical variables were analyzed using the Fisher exact test and are shown as numbers with percentages. Continuous variables were analyzed using the Mann-Whitney U test and are shown as the median and range. All *P* values were based on two-sided statistical tests, and the significance level was set at 0.05. The exact 95% confidence interval (CI) of risk difference was constructed. All the statistical analyzes were performed using JMP12.0.1 (SAS Institute, Cary, NC, USA).

This study was approved by the National Cancer Center Institutional Review Board (IRB) and by the IRBs of the other 7 participating institutions.

## Results

### Clinicopathological characteristics

Table 1 shows the clinical variables of the 29 patients who were studied. Nine patients (31%) had BR-PV, and 19 patients (66%) had BR-SMA (overlap allowed). The overall tumor size decreased from 29 mm to 26 mm after NACRT. The overall CA 19–9 level also exhibited a sharp decrease from 202.2 U/mL to 68.7 U/mL. A radiologic tumor response to NACRT with S-1 was observed in 3 patients (10%). There were 3 patients with partial responses (PRs; 10%), 25 with stable diseases (SDs; 86%), and 1 with PD (3%) according to the RECISTv1.1 criteria.

**Table 1:** Clinical characteristics of patients with borderline resectable pancreatic cancer

Preoperative variables	*N* = 29
Age, years	66 [50–78]
Sex (male)	19 (66%)
ECOG-PS (0/1)	27 (93%)/2 (7%)
Tumor location (Ph/Pb)	24 (83%)/5 (17%)
UICC 7th, Stage (I/II/III)	0/9 (31%)/20 (69%)
Type of tumor-vessel contact	
BR-PV	9 (31%)
BR-SMA	19 (66%)
BR-CHA	3 (10%)
BR-CeA	1 (3%)
Tumor size, mm	
Before NACRT	29 [17–40]
After NACRT	26 [11–38]
CEA, ng/mL	
Before NACRT	3.5 [1.1–28.4]
After NACRT	3.3 [1.5–18.0]
CA 19–9, U/mL	
Before NACRT	202.2 [1.0–4626.0]
After NACRT	68.7 [0.6–1530.0]
RECIST classification (PR/SD/PD)	3 (10%)/25 (86%)/1 (3%)
Operative variables	*N* = 29
Surgical procedure	
Pancreaticoduodenectomy	24 (83%)
Distal pancreatectomy	5 (17%)
Portal vein resection	16 (55%)
Partial resection of SMA plexus	27 (93%)
Number of retrieved lymph nodes	21 [5–51]
Blood loss, ml	835 [19–2589]

*ECOG-PS* Eastern Cooperative Oncology Group performance status, *Ph* Pancreas head, *Pb* pancreas body, *UICC 7th* The Union for International Cancer Control (UICC) classification 7th edition, *BR* Borderline resectable, *PV* Portal vein, *SMA* Superior mesenteric artery, *CHA* Common hepatic artery, *CeA* Celiac artery, *NACRT* Neoadjuvant chemoradiotherapy; RECIST, response evaluation criteria in solid tumors; PR, partial response; SD, stable disease, *PD* Progressive disease, *CEA* Carcinoembryonic antigen, *CA 19–9* Carbohydrate antigen 19–9

Table 2 shows the pathological variables among the patients. In terms of the residual tumor status, 27 (93%) of the 29 patients underwent R0 resection, and one each underwent R1 or RX resections, respectively. RX resection means that the resection margin could not be evaluated. R2 resection, probe laparotomy, and by-pass operations were not performed. The destruction of more than 50% of the tumor cells (grade ≥ IIb according to the Evans classification) was observed in 8 patients (28%).

**Table 2:** Pathological variables of patients with borderline resectable pancreatic cancer

	*N* = 29
Tumor size, mm	30 [10–40]
Lymph node metastasis	11 (38%)
Portal vein invasion	10 (34%)
Plexus invasion	12 (41%)
Stage (I/II/III)	3 (10%)/26 (90%)/0
Residual tumor (R0/R1/RX)	27 (93%)/1 (3%)/1 (3%)
Evans grade (I/IIa/IIb/III/IV)	5 (17%)/16 (55%)/4 (14%)/4 (14%)/0

Data are presented as *n* (%) or median [range]

### R0 resection rates based on the imaging findings of peripancreatic vascular invasion and tumor response

**Fig. 1:**
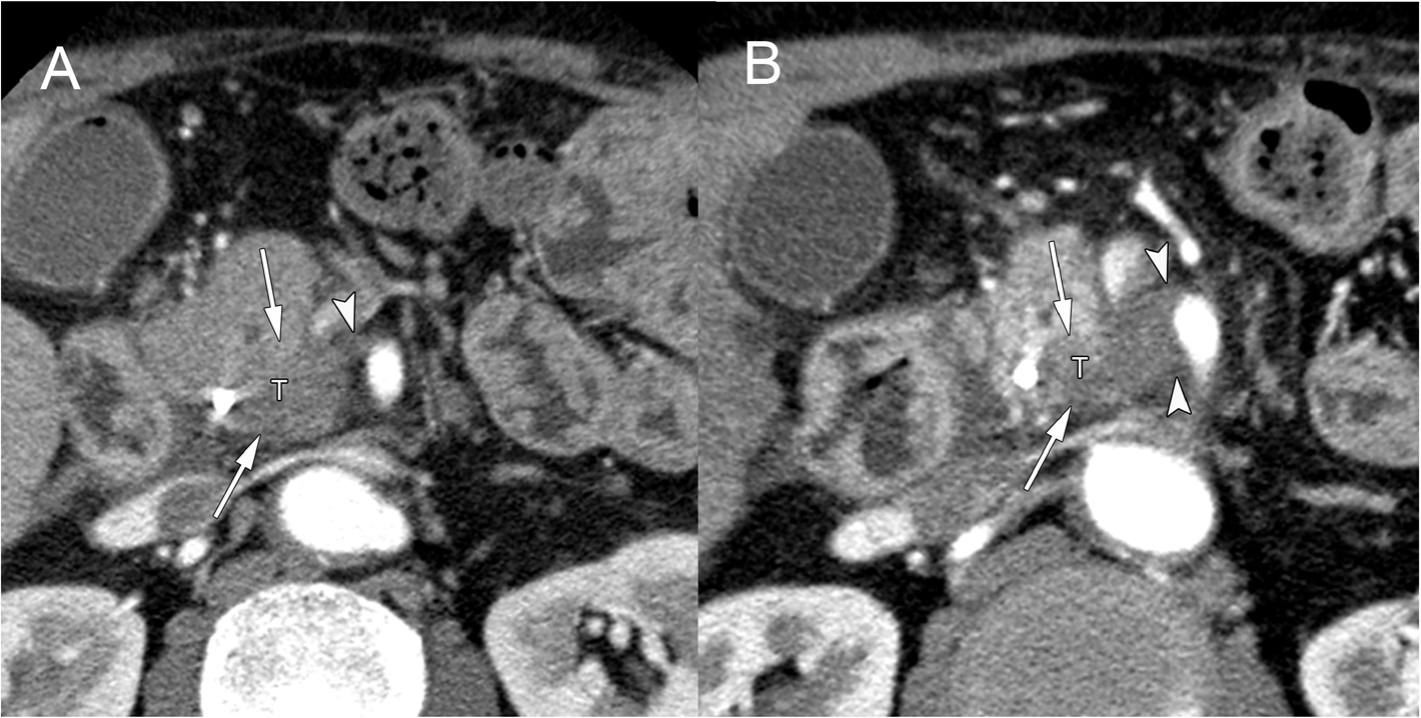
A patient with borderline resectable pancreatic cancer (**a**) before and (**b**) after neoadjuvant chemoradiotherapy. Legend; The pancreatic cancer in the uncinate process involved the superior mesenteric artery (SMA) and the superior mesenteric vein. The primary tumor (T) showed shrinkage (arrows), but the vascular invasion of the SMA (arrowheads) showed continued progression after neoadjuvant S-1 and concurrent radiotherapy

Nine patients (31%) were classified as PVI, and 20 (69%) were classified as NVI. All the patients in the PVI group underwent an R0 resection (9/9; R0 resection rate = 100%), while 90% (18/20) of the patients in the NVI group underwent an R0 resection. The exact 95% CI of risk difference between the PVI and NVI groups was − 10.0% [− 31.7–20.4%]. Furthermore, patients were classified into 6 groups according to the CT imaging findings for peripancreatic vascular invasion and tumor response according to the RECIST (Table 3). The R0 resection rates were not different among these 6 groups either. Figure 1 presents a representative patient from the PVI group in whom an R0 resection was achieved.

**Table 3:** R0 resection rate according to RECIST classification and vascular invasion before and after NACRT

		Vascular invasion	
		NVI group (*n* = 20)	PVI group (*n* = 9)
RECIST classification	PR	3/3 (100%)	0
	SD	^a^15/17 (88%)	8/8 (100%)
	PD	0	1/1 (100%)
Total		^a^18/20 (90%)	9/9 (100%)

*NVI* Non-progression of vascular invasion (shrinkage or stable), *PVI* Progression of vascular invasion, *RECIST* Response evaluation criteria in solid tumors, *PR* Partial response, *SD* Stable disease, *PD* Progressive disease

^a^One patient underwent R1 resection, and 1 patient underwent RX resection

### Relationships between grade of peripancreatic major vessel invasion and other evaluation items

Table 4 shows the rates of reduction in CEA and CA 19–9 after NACRT, the tumor responses according to RECIST, and the pathological responses in both the NVI and PVI groups. None of the aforementioned factors differed significantly between the PVI and NVI groups.

**Table 4:** Relationship between peripancreatic vascular invasion and clinicopathological variables on CT imaging

	NVI group (*n* = 20)	PVI group (*n* = 9)	*P* value
R0 resection rate	18 (90%)	9 (100%)	1.000
Change in CEA (%)	+ 5% [−51 − + 138]	−4% [− 54 − + 140]	0.572
Change in CA 19–9 (%)	− 45% [−94 − + 82]	−58% [−85– − 13]	0.278
RECIST classification (PR/SD/PD)	3 (15%)/17 (85%)/0	0/8 (89%)/1 (11%)	0.167
Evans grade ≥ IIB	6 (30%)	2 (22%)	1.000

*PVI* Progression of vascular invasion, *NVI* Non-progression of vascular invasion, *CEA* Carcinoembryonic antigen, *CA 19–9* Carbohydrate antigen 19–9, *RECIST* Response evaluation criteria in solid tumors, *PR* Partial response, *SD* Stable disease, *PD* Progressive disease. Data are presented as *n* (%) or median [range]

### Factors associated with pathological response of Evans grade ≥ IIb

Twenty-one patients were classified as having a pathological response of Evans grade ≤ IIa; 8 were classified as having Evans grade ≥ IIb. When these patients were compared, neither the rate of decrease in tumor markers (CEA or CA 19–9) after NACRT, the grade of peripancreatic major vessel invasion, nor the tumor response according to the RECIST differed significantly (Table 5). As a result, no evaluation factor was determined to be associated with the pathological response.

**Table 5:** Relationship between Evans grade and clinicopathological variables

	Evans grade ≤ IIa (*n* = 21)	Evans grade ≥ IIb (*n* = 8)	*P* value
Change in CEA (%)	+ 6% [− 45 − + 140]	−4% [− 54 − + 41]	0.180
Change in CA 19–9 (%)	− 45% [− 85 − + 82]	− 52% [− 94– − 13]	0.262
Change in vascular invasion			
shrinkage/stable, progression	4 (19%)/17 (81%)	4 (50%)/4 (50%)	0.164
shrinkage, stable/progression	14 (67%)/7 (33%)	6 (75%)/2 (25%)	1.000
RECIST classification (PR/SD/PD)	2 (10%)/19 (90%)/0	1 (13%)/6 (75%)/1 (13%)	0.242

*CEA* Carcinoembryonic antigen, *CA 19–9* Carbohydrate antigen 19–9, *RECIST* Response evaluation criteria in solid tumors, *PR* Partial response, *SD* Stable disease, *PD* Progressive disease. Data are presented as *n* (%) or median [range]

## Discussion

The present study showed that the response of peripancreatic vascular invasion after NACRT did not reflect the tumor response according to the RECIST. In the 29 BRPC patients, 9 (31%) experienced the progression of peripancreatic vascular invasion after NACRT, whereas local progression corresponding to PD according to the RECIST was only observed in 1 patient (3%). Moreover, the response of peripancreatic vascular invasion was likely to be uncorrelated with the actual resectability, since a high rate of R0 resection was achieved not only in the NVI group (90%), but also in the PVI group (100%) with the risk difference of − 10% [− 31.7–20.4%]. The progression of vascular invasion also seemed unrelated to the pathological response. These results would support our hypothesis that the radiological progression of the perivascular component of the tumor after NACRT might not reflect actual progression if the primary tumor does not exhibit progressive disease.

Patients with BRPC have a greater risk of being unable to receive an R0 resection than those with resectable pancreatic cancer, even when vascular resection and reconstruction are utilized [16]. Currently, BRPC is treated using a multimodality approach, usually consisting of neoadjuvant systemic therapy with or without radiotherapy to sterilize the tumor boundaries that are in contact with the peripancreatic vessels to enable a successful R0 resection [17–19].

Several studies have highlighted the difficulty in assessing local tumor extension using CT after NACRT for pancreatic cancer [20–22]. Cassinotto et al. found that 39% of patients who had a high risk of an R1 resection based on their CT findings after neoadjuvant therapy were able to receive an R0 resection. They surmised that the significantly lower accuracy and specificity in determining R0 resectability after neoadjuvant therapy was probably due to the inability of CT examinations to differentiate tumor tissue from necrosis, fibrosis, and inflammatory changes caused by neoadjuvant treatment.

Katz et al. demonstrated that 81 of the 85 patients (95%) received an R0 resection after being treated with neoadjuvant therapy for BRPC. In that study, only 1 patient was downstaged to resectable pancreatic cancer [23]. Ferrone et al. also demonstrated that 92% of patients with borderline resectable or locally advanced pancreatic cancer after receiving the neoadjuvant FOLFIRINOX regimen received an R0 resection regardless of having been judged as being unresectable based on the post-FOLFIRINOX CT imaging findings [24]. Both studies showed that downstaging to resectable pancreatic cancer from the CT imaging findings was rare after neoadjuvant therapy; however, R0 resection could be performed regardless of the radiological response when no distant metastatic lesions were observed.

In most of the resectability criteria, the extent of tumor-vessel contact was evaluated according to the degree of circumferential tumor–vessel interface. In the present study, however, we tried to assess tumor-vessel contact by the longitudinal distance of tumor–vessel interface to observe more detailed morphological changes. Specifically, we defined the progression of peripancreatic major vessel invasion as an increase in the length of tumor-vessel contact ≥2 mm because this definition enabled a good agreement with the interpreting physicians’ impressions of progression. Using this definition, 31% of the BRPC patients showed the progression of peripancreatic major vessel invasion after NACRT. However, 93% of the BRPC patients were able to receive an R0 resection irrespective of their peripancreatic major vessel invasion status. Furthermore, the peripancreatic major vessel invasion status did not seem correlated with any of the other items that were used to evaluate the efficacy of neoadjuvant treatment, such as the rate of reduction in CA 19–9, the tumor response, or the pathological response. These results would agree with the results of the aforementioned studies and lead us to conjecture that BRPC can be resected locally whether or not radiological perivascular progression is observed whenever the tumor response is not PD according to the RECIST. However, the surgical indications for BRPC with a PD tumor response remain uncertain because only one patient who met the above condition was included in the present study.

The present study had several limitations. First, the retrospective design introduced a selection bias. Several patients who underwent a probe laparotomy or bypass operation because of local tumor progression were excluded because the institutions where the patients had undergone these treatments did not participate in this study. This selection bias might have affected the results of the study. Second, the number of patients included in this study was relatively small, even considering the rarity of this disease. The small sample size probably had an affect on the wide CI of the risk difference of R0 resection rates between PVI and NVI groups. However, those R0 resection rates themselves were acceptable. Then, the conclusions which were drawn from the results including the R0 resection rates might be considered of value. Validation of the study’s hypothesis in a larger cohort is warranted.

## Conclusions

Patients with BRPC achieved a high R0 resection rate after NACRT regardless of the progression of peripancreatic vascular invasion. Patients with BRPC without PD according to the RECIST after NACRT can be considered as candidates for surgical resection.

## Data Availability

We promise to that the materials described in the manuscript, including all relevant raw data, will be freely available to any scientist wishing to use them for non-commercial purposes, without breaching participant confidentiality. The datasets used and/or analyzed during the current study are available from the corresponding author on reasonable request.

## Abbreviations

BRPC: Borderline resectable pancreatic cancer
MDCT: Multidetector computed tomography
NACRT: Neoadjuvant chemoradiotherapy
PVI: Progression of vascular invasion
NVI: Non-progression of vascular invasion
NCCN: National Comprehensive Cancer Network
NAC: Neoadjuvant chemotherapy
NCCHE: National Cancer Center Hospital East
ECOG: Eastern Cooperative Oncology Group
UICC: Union for International Cancer Control
CEA: Carcinoembryonic antigen
CA 19–9: Carbohydrate antigen 19–9
RECIST: Response evaluation criteria in solid tumors classification
SMA: Superior mesenteric artery
PD: Progressive disease
PV: Portal vein
CHA: Hepatic artery
CeA: Celiac artery
